# Multi-Parametric MRI and Texture Analysis to Visualize Spatial Histologic Heterogeneity and Tumor Extent in Glioblastoma

**DOI:** 10.1371/journal.pone.0141506

**Published:** 2015-11-24

**Authors:** Leland S. Hu, Shuluo Ning, Jennifer M. Eschbacher, Nathan Gaw, Amylou C. Dueck, Kris A. Smith, Peter Nakaji, Jonathan Plasencia, Sara Ranjbar, Stephen J. Price, Nhan Tran, Joseph Loftus, Robert Jenkins, Brian P. O’Neill, William Elmquist, Leslie C. Baxter, Fei Gao, David Frakes, John P. Karis, Christine Zwart, Kristin R. Swanson, Jann Sarkaria, Teresa Wu, J. Ross Mitchell, Jing Li

**Affiliations:** 1 Department of Radiology, Mayo Clinic, Phoenix, Arizona, United States of America; 2 Department of Biostatistics, Mayo Clinic, Phoenix, Arizona, United States of America; 3 Department of Neurosurgery, Mayo Clinic, Phoenix, Arizona, United States of America; 4 Department of Cancer and Cell Biology, Mayo Clinic, Scottsdale, AZ, United States of America; 5 Department of Pathology, Mayo Clinic, Rochester, Minnesota, United States of America; 6 Department of Neuro-oncology, Mayo Clinic, Rochester, Minnesota, United States of America; 7 Department of Radiation Oncology, Mayo Clinic, Rochester, Minnesota, United States of America; 8 Department of Pharmacology, University of Minnesota, Minneapolis, Minnesota, United States of America; 9 Department of Cancer and Cell Biology, Translational Genomics Research Institute, Phoenix, Arizona, United States of America; 10 School of Computing, Informatics and Decision Systems Engineering, Arizona State University, Tempe, Arizona, United States of America; 11 School of Biological and Health Systems Engineering, Arizona State University, Tempe, Arizona, United States of America; 12 Department of Pathology, Barrow Neurological Institute, Phoenix, Arizona, United States of America; 13 Department of Neurosurgery, Barrow Neurological Institute, Phoenix, Arizona, United States of America; 14 Department of Radiology, Barrow Neurological Institute, Phoenix, Arizona, United States of America; 15 Department of Clinical Neurosciences, University of Cambridge, Cambridge, United Kingdom; Banner Alzheimer's Institute, UNITED STATES

## Abstract

**Background:**

Genetic profiling represents the future of neuro-oncology but suffers from inadequate biopsies in heterogeneous tumors like Glioblastoma (GBM). Contrast-enhanced MRI (CE-MRI) targets enhancing core (ENH) but yields adequate tumor in only ~60% of cases. Further, CE-MRI poorly localizes infiltrative tumor within surrounding non-enhancing parenchyma, or brain-around-tumor (BAT), despite the importance of characterizing this tumor segment, which universally recurs. In this study, we use multiple texture analysis and machine learning (ML) algorithms to analyze multi-parametric MRI, and produce new images indicating tumor-rich targets in GBM.

**Methods:**

We recruited primary GBM patients undergoing image-guided biopsies and acquired pre-operative MRI: CE-MRI, Dynamic-Susceptibility-weighted-Contrast-enhanced-MRI, and Diffusion Tensor Imaging. Following image coregistration and region of interest placement at biopsy locations, we compared MRI metrics and regional texture with histologic diagnoses of high- vs low-tumor content (≥80% vs <80% tumor nuclei) for corresponding samples. In a training set, we used three texture analysis algorithms and three ML methods to identify MRI-texture features that optimized model accuracy to distinguish tumor content. We confirmed model accuracy in a separate validation set.

**Results:**

We collected 82 biopsies from 18 GBMs throughout ENH and BAT. The MRI-based model achieved 85% cross-validated accuracy to diagnose high- vs low-tumor in the training set (60 biopsies, 11 patients). The model achieved 81.8% accuracy in the validation set (22 biopsies, 7 patients).

**Conclusion:**

Multi-parametric MRI and texture analysis can help characterize and visualize GBM’s spatial histologic heterogeneity to identify regional tumor-rich biopsy targets.

## Introduction

Glioblastoma (GBM) represents one of the most genetically heterogeneous, resistant, and lethal of all human cancers [1,2]. While median survival remains poor with conventional therapy, the use of genomic profiling has ushered promising new approaches to drug discovery and treatment optimization [3]. Recent landmark initiatives by the National Cancer Institute (NCI) and The Cancer Genome Atlas (TCGA) have sought to catalog GBM’s diverse genetic landscape, giving insight to pathogenesis, prognosis and therapeutic susceptibility. This should help guide risk stratification for existing protocols and help identify key driver genes as potential therapeutic targets in the future [4]. With improving availability and cost, genomic profiling will play an ever-increasing role in the paradigm of individualized oncology.

Yet, securing tumor-rich biospecimens for genomic profiling remains a significant challenge. In their initial report, TCGA found that only 35% of submitted biopsy samples contained adequate tumoral content and/or genetic material [4]. This low yield relates to GBM’s profound histologic heterogeneity and the limitations of contrast-enhanced MRI (CE-MRI)-guided biopsies to distinguish enhancing tumor from non-tumoral tissue (e.g., reactive gliosis, microscopic necrosis). CE-MRI also poorly localizes tumor within surrounding non-enhancing parenchyma, or Brain Around Tumor (BAT), which appears indistinguishable from non-tumoral vasogenic edema [5]. Finally, recently proposed multisampling approaches, which help characterize intratumoral heterogeneity, further increase the risk of low tumor content because biopsy volumes are typically smaller than en bloc resection [6].

Advanced MRI offers an adjunct to conventional imaging and should help delineate tumor-rich biopsy targets. Image-based features such as tumor cell density on diffusion-weighted imaging (DWI), white matter infiltration on diffusion tensor imaging (DTI), and microvessel morphology on perfusion MRI (pMRI) reflect key biophysical characteristics associated with tumor pathogenesis [5,7–10]. Additionally, textural patterns between spatially encoded voxels and their surrounding neighbors provide further insight to regional microstructure and histologic identity [11–14]. And while most imaging techniques have been evaluated individually, the multi-parametric nature of MRI enables co-localization and incorporation of multiple complementary features to optimize diagnostic accuracy [15]. Multiple texture algorithms offer similar advantages to using multiple MR contrasts—we expect each algorithm to provide additional information.

Although prior studies suggest benefits from combining imaging techniques, there is currently no system that integrates the diverse image-based phenotypes and rich textural data of multi-parametric MRI to quantify tumor content within biopsy targets. To address this need, we assess the feasibility of using machine learning (ML) to integrate an array of image-based texture features from pre-operative MRI to predict tumor-rich biopsies from both enhancing core and non-enhancing BAT in a cohort of GBM patients. Our overarching goal is to develop non-invasive correlates of histology that can facilitate image-guided biopsy and genomic profiling within the framework of individualized oncology.

The unique contributions of this study are: 1) a new method to produce images of histologic tumor content within an individual GBM; 2) a new protocol that uses multiple machine learning and texture algorithms to analyze co-registered multi-parametric MRI; 3) model construction and cross-validation in a training cohort of patients, followed by validation in a de-novo group of patients; and, 4) a model with high accuracy predicting histologic tumor content in the non-enhancing, and traditionally problematic, brain-around-tumor (BAT) zone.

## Methods

### Patient recruitment

We recruited patients with clinically suspected GBM undergoing preoperative stereotactic MRI for surgical resection. We confirmed the absence of previous treatment (including steroid administration) and excluded subjects with an estimated glomerular filtration rate <60 mg/min/1.72 m2. We obtained approval from the institutional review boards at Barrow Neurological Institute (BNI) and Mayo Clinic in Arizona (MCA) and obtained written and informed consent from each subject prior to enrollment.

### Surgical biopsy

Our group used pre-operative conventional MRI, including T1-Weighted contrast-enhanced (T1+C) and T2-Weighted sequences (T2W), to guide stereotactic biopsies as previously described [16]. In short, each neurosurgeon collected an average of 5–6 tissue specimens from each tumor by using stereotactic surgical localization, following the smallest possible diameter craniotomies to minimize brain shift. Neurosurgeons selected targets separated by at least 1 cm from both enhancing core (using T1+C) and non-enhancing BAT (using T1+C and/or T2W) in pseudorandom fashion, typically from different poles of the enhancing lesion periphery while avoiding any necrotic regions, based on clinical feasibility as per clinical protocol. The neurosurgeons recorded biopsy locations via screen capture to allow subsequent coregistration with multiparametric MRI datasets. The neurosurgeon visually validated stereotactic imaging locations with corresponding intracranial anatomic landmarks, such as vascular structures and ventricle margins, before recording specimen locations.

### Histologic analysis and biopsy sample classification

Tissue specimens (target volume of 125mg) were flash frozen in liquid nitrogen within 1–2 min from collection in the operating suite and stored in -80°C freezer until subsequent processing. Tissue was retrieved from the freezer and embedded frozen in optimal cutting temperature (OCT) compound. Tissue was cut at 4 um sections in a -20 degree C cryostat (Microm-HM-550) utilizing microtome blade. Tissue sections were stained with hematoxylin and eosin (H&E) for neuropathology review. H&E slides were reviewed blinded to diagnosis by a neuropathologist and assessed for tumor content. Taking into account all visible cells (neurons, inflammatory cells, reactive glia, tumor cells, etc.), the percent tumor nuclei were estimated. Based on the TCGA criteria, we used the threshold of at least 80% tumor nuclei content to define tumor-rich (i.e., high tumor) biopsy samples [4]. Those with less than 80% tumor content were classified as low tumor samples.

### MRI protocol, parametric maps, and image coregistration

#### Conventional MRI and general acquisition conditions

We performed all imaging on a 3 Tesla system (Sigma HDx; GE-Healthcare, Milwaukee, Wisconsin) within 1 day prior to stereotactic surgery. Conventional MRI included standard pre- and post-contrast T1-Weighted (T1-C, T1+C, respectively) and pre-contrast T2-Weighted (T2W) sequences. T1W images were acquired using spoiled gradient recalled-echo inversion-recovery prepped (SPGR-IR prepped) (TI/TR/TE, 300/6.8/2.8ms; matrix, 320×224; FOV, 26 cm; thickness 2mm). T2W images were acquired using fast-spin-echo (FSE) (TR/TE, 5133/78ms; matrix 320x192; FOV 26cm; thickness 2mm). T1+C images were acquired after completion of Dynamic Susceptibility-weighted Contrast-enhanced (DSC) Perfusion MRI (pMRI) following total Gd-DTPA (gadobenate dimeglumine) dosage of 0.15 mmol/kg as described below [16,17].

#### Diffusion Tensor (DTI)

DTI imaging was performed using Spin-Echo Echo-planar imaging (EPI) [TR/TE 10000/85.2ms, matrix 256x256; FOV 30cm, 3mm slice, 30 directions, ASSET, B = 0,1000]. The original DTI image DICOM files were converted to a FSL recognized NIfTI file format, using MRIConvert (http://lcni.uoregon.edu/downloads/mriconvert), before processing in FSL from semi-automated script. In total, 12 DTI parametric maps were calculated using FSL (http://fsl.fmrib.ox.ac.uk/fsl/fslwiki/), including: isotropic (p) and anisotropic (q) diffusion, mean diffusivity (MD) and fractional anisotrophy (FA) based on previously published methods [5].

#### DSC-pMRI

Prior to DSC acquisition, preload dose (PLD) of 0.1 mmol/kg was administered to minimize T1W leakage errors. After PLD, we employed Gradient-echo (GE) EPI [TR/TE/flip angle = 1500ms/20ms/60°, matrix 128x128, thickness 5mm] for 3 minutes. At 45 sec after the start of the DSC sequence, we administered another 0.05 mmol/kg i.v. bolus Gd-DTPA [16,17]. The initial source volume of images from the GE-EPI scan contained negative contrast enhancement (i.e., susceptibility effects from the PLD administration) and provided the MRI contrast labeled EPI+C. At approximately 6 minutes after the time of contrast injection, the T2*W signal loss on EPI+C provides information about tissue cell density from contrast distribution within the extravascular, extracellular space [18]. We performed leakage correction and calculated relative cerebral blood (rCBV) based on the entire DSC acquisition using IB Neuro as referenced [17]. We also normalized rCBV values to contralateral normal appearing white matter as previous described [16,17].

#### Image coregistration

For image coregistration, we employed tools from ITK (www.itk.org) and IB Suite (Imaging Biometrics) as previously described [17]. Because the majority of MRI contrasts (4 out of 8) originated from the DTI acquisition, we chose to coregister all datasets to the relatively high quality DTI B0 anatomical image volume. This offered the additional advantage of minimizing potential distortion errors (from data resampling) that could preferentially impact the mathematically sensitive DTI metrics. This also avoided upsampling of advanced MRI maps to the relatively high resolution of T1W stereotactic data (particularly when acquired with 512x512 matrix), which could result in textural artifacts during image processing. Ultimately, the coregistered data used for texture analysis exhibited in plane voxel resolution of ~1.2 mm (256x256 matrix) and slice thickness of 3mm.

### Texture analysis and image processing

A board-certified neuroradiologist (LH) placed square (8x8 voxel) regions of interest (ROIs) at the locations corresponding to each biopsy site. For all analysis methods, we extracted ROIs from original images and analyzed each independently. Prior to texture analysis, we acquired first order statistics from raw image signals: average and standard deviation (SD) of gray-level intensities. Next, intensity values within each extracted ROI were mapped onto the range 0–255. This step helped standardize intensities between ROIs and reduced effects of intensity non-uniformity on features extracted during subsequent texture analysis. In this study, we incorporate 3 separate but complementary texture algorithms with multi-parametric MRI to characterize GBM’s regional variability in histologic tumor content [11–14]. Multiple texture algorithms offer similar advantages to using multiple MR contrasts—we expect each algorithm to provide additional information. We applied each of the three texture analysis methods for each ROI, which generated a total of 30 texture features for each of 8 total MRI contrasts (T1+C, T2W, rCBV, EPI+C, p, q, MD, FA). Therefore, at each coregistered ROI, we calculated a total of 240 texture features, in addition to the 16 raw features (i.e., mean, SD for 8 MRI contrasts), yielding a total of 256 image-based features for classification. The 3 texture analysis methods are described briefly below.

#### Gray Level Co-Occurrence Matrix (GLCM)

GLCM provides detailed gray scale data by describing the angular relationships and distances between neighboring image voxels with similar gray scale intensities [11]. Commonly used in texture analysis, GLCM uses second order statistics of the distribution of gray-scale intensity level within a ROI. Each element in the co-occurrence matrix shows how often a pair of intensity levels is seen in a configuration defined by a certain radius and angle. The co-occurrence matrix was computed by averaging over four uniformly distributed angular directions (0^0^, 45^0^, 90^0^, and 135^0^) to produce a set of 13 rotationally invariant features: entropy (matrix randomness), energy/angular second moment (pixel repetition/orderliness, measures image homogeneity), homogeneity (uniformity of co-occurrence matrix), dissimilarity (measurement of how different each matrix element is), and correlation (measurement of gray-tone linear dependencies) [11]. In total, we calculated 13 GLCM texture features at each ROI for each MRI contrast.

#### Local Binary Patterns (LBP)

LBP provides highly discriminatory rotational and illumination invariant structural information by labeling each image voxel (in binary fashion) as higher or lower intensity compared with neighboring voxels [12]. Highly cited as a method for texture description, LBP evaluates the intensity distribution of the set of points within a certain radius of each voxel in the ROI. Local binary numbers are categorized into ‘uniform’ and ‘non-uniform’ patterns based on the number of bit-wise transitions from 0 to 1 or visa versa. A histogram of the labels is used as a measure of uniformity for the ROI. We used the neighborhood radius of three voxels (the 24 points circularly surrounding each voxel) as the ROI for our experiments. Given the 8x8 voxel ROI size, the 3-voxel radius represents the maximum for confining analysis within the ROI while providing larger scale pattern analysis. We chose not to use a smaller radius (e.g., 1-voxel or 2-voxel) because these provide smaller scale patterns that are already provided by GLCM. The value of bins in a 12-bin histogram was reported as the set of feature for this method. In total, we calculated 12 LBP texture features at each ROI for each MRI contrast.

#### Discrete Orthonormal Stockwell Transform (DOST)

DOST directly measures local spatial frequencies, which have shown specific applicability to neurologic diseases such as glioma [13,14]. The DOST provides a complete minimal multi-resolution spatial-frequency representation of an image. It maintains the phase information while avoiding redundant calculations of time-frequency information and thus being computationally less expensive. The two-dimensional DOST uses a dyadic sampling scheme (orders 0,1,2…Log (N)-1) to partition the 2D Fourier transform of the image into non-overlapping sections. Each section, including only a band-limited subspace of the FT domain, is shifted in frequency and phase to produce the DOST image. Averaging DOST elements in each section results in a harmonics image from which we calculated rotationally invariant features [13,14]. In total, we calculated 5 DOST features at each ROI for each MRI contrast.

### Principal Component Analysis (PCA) for dimension reduction

With a total of 256 image-based features from each ROI and corresponding biopsy sample, the dimensionality of the image-based features far exceeded the sample size, increasing the risk of overfitting artifacts and creating challenges for classification. To address this, we used Principal Component Analysis (PCA) which identifies the linear combination of features, called Principal Components (PCs), to reduce the dimensionality of the imaging data [19–21]. Here we identified the PCs separately for each MRI contrast and texture algorithm for better clinical interpretation. Usually, between 1–3 PCs sufficiently account for the variability in the original feature space, so we retained those PCs that accounted for 85% of original feature variability for further analysis [19–21].

### Classification with sequential forward feature selection

We used classification algorithms with sequential forward feature selection to identify the subset of image-based PCs (determined from PCA above) with the greatest combined discrimination for biopsy tumor content (high- vs low-tumor). To represent the spectrum of classification methodologies, we separately applied 3 distinct but commonly described classification algorithms: Diagonal Linear Discriminate Analysis (DLDA), Diagonal Quadratic Discriminate Analysis (DQDA) and Support Vector Machines (SVM) [22–24]. As the names imply, DLDA identifies linear classification boundaries to separate classes, while DQDA identifies quadratic boundaries. SVM can identify complex boundaries for class separation [22–24]. We determined classification accuracy using leave-one-out cross validation (LOOCV). With LOOCV, all samples but one are used to develop the classifier (i.e., training set) with one randomly chosen sample serving as the test case for classification performance [13,14]. This process repeats for all samples in the dataset (i.e., 60 separate trial runs in our cohort), until each sample in the cohort has served as the test sample. The averaged accuracy is the overall cross validation (CV) accuracy. In building the classification model, the PCs are selected using sequential forward feature selection, which identifies the PC with greatest discriminatory power (e.g. PC1), then evaluates and adds additional PCs that contribute incremental gains to classification accuracy [19,20]. The iterative process continues to add remaining PCs to the classification model until accuracy gains cease (defined as incremental gain of <1%). Of all ML methods, DLDA identified the highest CV accuracy with the fewest required PCs (**S1 Appendix**). Thus, we henceforth report the results from the DLDA analysis.

## Results

### Training and Validation biopsy datasets

In total, we collected 82 biopsy samples from 18 GBM patients. We describe the distribution of training (60 biopsies, 11 patients) and validation (22 biopsies, 7 patients) biopsy samples throughout enhancing core (ENH) and BAT in **Table 1**. The vast majority of biopsies (90%) were separated by >1cm. Overall, 59.2% of tissue samples from ENH demonstrated high tumor content, similar to the frequency of adequate tumor samples reported by the TCGA (60.2%). When samples originated from BAT, a much lower percentage (21.2%) of samples demonstrated high tumor content.

**Table 1:** Summary of tissue samples and test performance in both training and validation datasets.

	Training Set			Validation Set		
	(n = 11 subjects)			(n = 7 subjects)		
	ENH	BAT	Both	ENH	BAT	Both
**Total samples**	35	25	60	14	8	22
**High tumor samples**	22	5	27	7	2	9
**(% of total samples)**	(62.9%)	(20%)	(45%)	(50%)	(25%)	(41%)
**Low tumor samples**	13	20	33	7	6	13
**(% of total samples)**	(37.1%)	(80%)	(55%)	(50%)	(75%)	(59%)
**Imaging accuracy(high vs low tumor)**	82.9%	88%	85.0%	78.6%	87.5%	81.8%
**Sensitivity(identify high-tumor)**	86.4%	80%	85.2%	100%	100%	100%
**Specificity(identify high tumor)**	76.9%	90%	84.8%	57.1%	83.3%	69.2%
**PPV(predict high tumor)**	86.4%	66.7%	82.1%	70%	66.7%	69.2%
**NPV(exclude high tumor)**	76.9%	94.7%	87.5%	100%	100%	100%

Distribution of biopsy samples by tumor content (high- vs. low-) for enhancing core (ENH) and non-enhancing BAT in both training and validation datasets. Test accuracies (sensitivity, specificity) for the optimized model (based on 3 MRI texture features) are shown and include positive and negative predictive values (PPV, NPV).

### Classification accuracy

**Table 2**lists selected features and the cross-validation (CV) accuracy for DLDA, DQDA and SVM. Specifically, DLDA with sequential forward feature selection, identified 3 MRI-based texture features that optimized the classification model to distinguish high- vs. low-tumor biopsy samples (85% accuracy). The three features are: raw measure of rCBV, GLCM feature from EPI+C and LBP feature from T1+C. In general, rCBV provides non-invasive measures of microvascular volume, which link closely to histologic tumor content, malignant potential, and prognosis [7,16,17]. The EPI+C MRI contrast (measured at ~ 5 minutes after contrast injection) measures negative enhancement (i.e., signal loss) that persists on static T2*W Gradient Echo images from Gd-DTPA extravasation and distribution within the extracellular space. This has been linked closely to tissue cellular density [18]. Finally, T1+C measures Gd-DTPA contrast enhancement from blood brain barrier (BBB) disruption and represents a secondary feature of tumor aggression [24].

**Table 2:** Summary of selected MRI-based texture features to optimize CV training accuracy.

MRI-based feature	Texture algorithm	Texture description	MRI Contrast	Physiologic correlate
**rCBV(raw mean)**	**–**	**–**	Relative cerebral blood volume (rCBV)	Micro-vessel volume
**EPI+C-GLCM**	Gray level co-occurrence matrix (GLCM)	Gray scale intensities	T2*W negative enhancement (EPI+C)	Tumor cell density
**T1+C-LBP**	Local binary product (LBP)	Structural uniformity	T1W contrast enhancement (T1+C)	BBB disruption

Machine learning (ML) selected the 3 MRI-based texture features that optimized cross validation (CV) accuracy based on leave-one-out cross validation (LOOCV) of the training set data (60 biopsies, 11 patients). The overall CV accuracy based on the 3 features is 85%.

**Fig 1:**
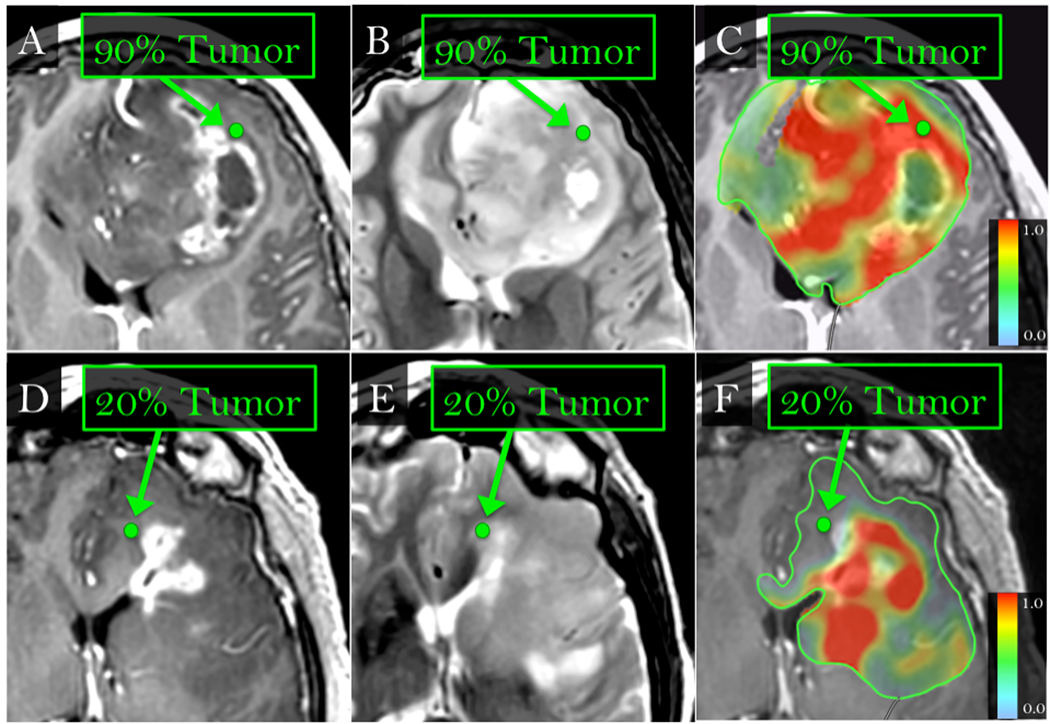
ML-based MRI invasion maps show tumor-rich (>80% tumor nuclei) extent throughout ENH and BAT. (A,B,C,E) Biopsy locations within the non-enhancing BAT zone (green dots, arrows) on T1+C (A,D) and T2W (B,E) images correspond with high-tumor (>80% tumor nuclei) and low-tumor (<80% tumor nuclei) tissue samples on histologic analysis. (C,F) Color overlay maps with manual tracings (green) around BAT show the probability (range 0–1) of tumor-rich (red) vs tumor-poor (green/blue) content, based on ML analysis and multi-parametric MRI in 60 training biopsies and 22 validation biopsies. The maps show correspondence between tumor-rich (B, red) and tumor-poor (D, blue/gray) biopsy samples.

After completing the MRI feature selection on the training dataset (n = 60), we applied the optimized model (using all three features) to classify high- vs. low-tumor content biopsies in a separate, retrospectively collected validation set (22 biopsies, 7 patients). The CV accuracy of the training set and the accuracy of applying the training model on the separate validation set are listed in **Table 1**for both enhancing core (ENH) and BAT. In the validation set, model classification achieved an overall accuracy (81.8%) similar to that in the training dataset. Importantly, the model achieved a high degree of accuracy in non-enhancing BAT (88% training, 87.5% validation), which represents a problematic area for conventional CE-MRI-guided biopsies [3,5,7,25]. Specifically, tumor-rich populations show low prevalence in the BAT (21.2% for all biopsies in our study) and typically remain indistinguishable from non-tumoral edema on CE-MRI [5]. The positive predictive values (PPV) of 66.7% in both training and validation sets suggests that using the MRI texture model would significantly improve the localization and recovery of tumor-rich BAT targets compared to current CE-MRI guided biopsy methods, as shown in **Fig 1**. Ten percent of training set biopsies (n = 6) were located within 5-10mm of adjacent biopsies. To test the potential confounding effects on accuracy, we performed a subanalysis that excluded these samples, as shown in **S2 Appendix**. With 54 total samples, the model achieved an accuracy of 85.2%, nearly identical to the main analysis, suggesting that the effects are negligible.

## Discussion

Genetic profiling represents the future of neuro-oncology but hinges on the recovery of adequate biopsy material from representative tumor segments [4,6]. While contrast-enhanced MRI (CE-MRI) serves as the current standard for image-guided surgery, distinct limitations exist. For instance, neurosurgeons routinely target CE-MRI enhancing core (ENH) for its presumed tumor-rich composition, but this yields adequate tumor in only ~60% of the cases from TCGA and our cohort [4]. Further, the BAT harbors residual populations that are most important to characterize because they universally recur, but the low prevalence of tumor-rich foci (~20% in our cohort) and the lack of enhancement in BAT limit the success of CE-MRI-guided biopsies [5,25]. In this study, we improve the detection of tumor-rich targets throughout ENH and BAT by combining CE-MRI with advanced imaging and texture analysis. Advanced MRI metrics on diffusion tensor imaging (DTI), diffusion weighted imaging (DWI), and perfusion MRI (pMRI) correlate with hallmark features of tumor aggression, such as white matter invasion (DTI), cellular proliferation (DWI), and tumoral angiogenesis (pMRI) [5,7–10]. Meanwhile, MRI texture–which characterizes regional heterogeneity and microstructure–can capture additional tissue features such as molecular status (i.e., MGMT, 1p/19q) and histologic identity [11–14,24].

Previous glioma studies have used advanced MRI-guided stereotactic biopsies to validate the correlations with spatially matched regional histology. Barajas et al. compared DWI and pMRI measurements with tumor cell density, among other histologic indices (e.g., microvascular hyperplasia, cell proliferation), in ENH and BAT biopsies [7]. Compared with DWI, they found that tumor cell density correlated more strongly with pMRI metrics, namely rCBV and relative peak height (rPH) in ENH samples, and rPH in BAT samples. Ellingson et al. determined the correlations between apparent diffusion coefficient (ADC, closely related to MD) on DWI and tumor cell density [8]. Similarly, LaViolette et al. confirmed the utility of ADC to identify regions of hypercellular non-enhancing tumor in ex-vivo cases, while Stadlbauer found FA to be more informative than MD (closely linked to ADC) [9,10]. Finally, Price et al. determined DTI thresholds for isotropic (p) and anisotropic (q) diffusion to distinguish normal white matter from tumoral infiltration and gross replacement [5]. While these studies have helped to establish advanced MRI-based correlates in glioma, none have tested the accuracy to classify biopsy targets based on quantitative thresholds for histologic tumor content (e.g., ≥80% vs <80% tumor nuclei). Further, we don’t yet know the diagnostic value of combining multiple complementary metrics or extracting regional image texture patterns from advanced MRI maps.

Based on the correlations in our training set, we have used machine learning (ML) to identify three MRI-based features that optimize classification accuracy for high- vs. low-tumor content (Table 2). Mean rCBV contributes the most to model accuracy, which aligns with previous pMRI studies showing strong correlations between rCBV and histologic tumor content, tumoral proliferation, and malignant potential. In particular, densely cellular tumor elaborates large microvascular networks (to support metabolic demands), which correspond to elevated rCBV values on pMRI [7,16,17]. Signal loss on T2*W EPI+C relates to contrast agent extravasation and subsequent equilibration within the extracellular space. Clinical and preclinical studies have shown how these susceptibility effects correlate strongly with tumor cell density and size, similar to using MD, ADC, and isotropic diffusion (p) on DWI and DTI [5,7,8–10]. Meanwhile, signal changes on T1+C represent regional differences in contrast extravasation (and signal rise) due to blood brain barrier (BBB) disruption. While T1+C routinely guides CE-MRI biopsies, our model shows that structural uniformity textures, rather than simply the presence/absence of T1+C enhancement, provide the greater contribution to test accuracy [12,24].

**Fig 2:**
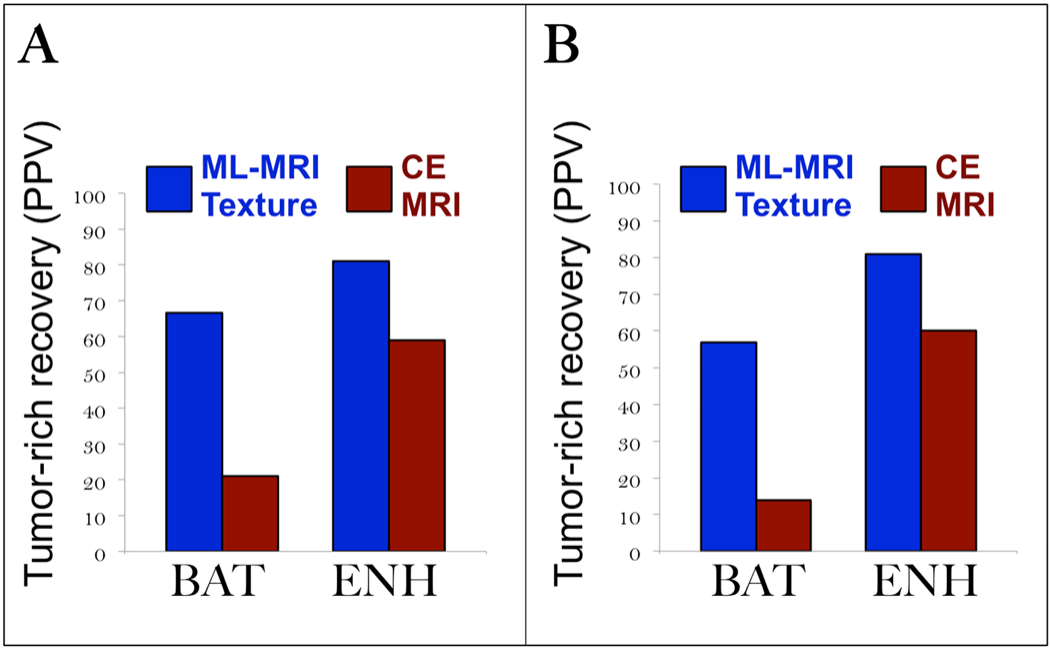
ML-based model improves tumor-rich biopsy delineation compared with CE-MRI. (A) ML-based MRI texture model in the full dataset (n = 82, Table 1) shows higher positive predictive values (PPV) (66.7% in BAT, 81.3% in ENH) for recovering tumor-rich samples compared with CE-MRI (21.2% in BAT, 59.2% in ENH). These PPVs suggest that the ML-based model would help recover tumor-rich BAT samples with over three times greater efficiency compared with CE-MRI guidance. (B) ML-based MRI texture model in the subanalysis (n = 76, S2 Appendix) provides higher positive predictive values (PPV) (57.1% in BAT, 80.6% in ENH) for recovering tumor-rich samples (>80% tumor nuclei) compared with CE-MRI (13.8% in BAT, 59.6% in ENH). Based on these PPVs, the ML-based model would enable four times more efficient tumor-rich recovery from BAT compared with CE-MRI guidance.

Overall model accuracy (85% training, 81.8% validation) suggests that the ML-based MRI texture model can guide surgical biopsies to improve recovery of tumor-rich biospecimens compared to current CE-MRI methods. Particularly encouraging is the high model accuracy for non-enhancing BAT (88% training, 87.5% validation). Even in the subgroup analysis, which excludes 6 samples within 5–10 mm of each other, the ML-based model achieves high accuracy in BAT (90.5%). Based on current limitations of CE-MRI and low prevalence of tumor-rich foci in BAT (21% in full cohort, 13.7% in subgroup analysis), neurosurgeons would need approximately 5–7 biopsy attempts to recover one adequate tumor-rich sample (≥80% tumor nuclei) [5,25]. The ML-based model’s PPV of ~67% significantly improves the efficiency of tumor-rich recovery in the problematic non-enhancing BAT zone, as illustrated in **Fig 2**. Also, the NPV (94.7% training, 100% validation) of the ML model would suggest a high degree of confidence in excluding tumor-rich populations in tumoral subregions, thereby improving surgical safety by preventing unnecessary biopsies. Besides biopsy guidance, the ML-based MRI texture model also presents applicability for improving the extent of maximal surgical resection, which correlates strongly with patient survival [26]. Currently, CE-MRI guides gross total resection (GTR) based on enhancing core (ENH), but this can leave substantial residual tumor burden in the non-enhancing BAT. Using the ML-based model to map tumor infiltration in BAT could help surgeons plan safer and more extensive resections to improve local control and patient outcomes. The study results here should provide the impetus and justification for future clinical trials that integrate this ML-based model into neuronavigational platforms to prospectively validate test accuracy and potential clinical impact.

A number of studies have shown the utility of intraoperative fluorescence microscopy with 5-aminolevulinic acid (5-ALA) to facilitate gross total resection (GTR) of enhancing core (ENH), which currently represents the primary target of surgical biopsy and resection [27,28]. The ENH tumor segment is well visualized on CE-MRI because of gadolinium-based contrast agent (GBCA) extravasation through a disrupted blood brain barrier (BBB). This BBB dependence also accounts for 5-ALA visualization of the ENH tumor segment, which may explain why both CE-MRI and 5-ALA poorly characterize the non-enhancing invasive tumor in BAT (which maintains BBB integrity) [27,28]. In fact, 5-ALA shows poor sensitivity for detection of invasive non-enhanicng tumor in BAT, with negative predictive value (NPV) as low as 26% [27]. For these reasons, the invasive tumor segment in BAT typically remains unresected, unbiopsied, and uncharacterized despite the use of CE-MRI and/or 5-ALA guidance. Neglecting BAT tumor at the time of surgery can leave substantial unresected tumor burden that contributes to recurrent disease. This could also have particular impact on the selection of adjuvant targeted therapies in the future, as we move towards the paradigm of individualized oncology, because the invasive tumor segment represents the focus of adjuvant therapy but can harbor different therapeutic targets than those detected in ENH biopsies [6,7,25]. Unless these BAT targets are biopsied and characterized, sampling error could misinform treatment, potentiating recurrence of pre-existing resistant clones. The ML-based model presented here helps to address this gap, by significantly improving the detection and recovery of tumor-rich biopsies in the problematic non-enhancing BAT zone (**Fig 2**).

In this study, we define high tumor content (≥80% tumor) based on published TCGA criteria for sample adequacy [4]. This threshold helps to maximize tumoral DNA quality by minimizing non-tumoral contamination. While our ML model demonstrates high accuracy to distinguish tumor content based on this specific histologic threshold, classification of samples based on other thresholds (e.g., 20%, 50%, 75% tumor nuclei) would require re-training of the ML model to maximize diagnostic accuracy. In other words, an ML model for one histologic threshold may not optimally translate to other histologic thresholds. This represents one of the limitations to using ML methodology. Nonetheless, additional ML models can be developed in future work for other clinically relevant histologic thresholds, or potentially for prediction of tumor cell density as a continuous variable (i.e., tumor nuclei range from 0–100%). We must also note that different histologic thresholds may favor specific MRI-based phenotypes. For instance, high tumor density may select for “proliferative” imaging phenotypes, while lower tumor thresholds (i.e., ≥ 50% or ≥20% tumor fraction) may associate with “invasive” phenotypes in which tumor admixes more evenly with surrounding non-tumoral parenchyma [5]. Thus, while DWI and DTI metrics were not selected in this study, they may prove useful when evaluating other tumor thresholds. Such thresholds may have greater applicability for image-guided interventions such as radiation therapy (RT), which generally targets both densely cellular tumor and more diffusely infiltrative tumor-poor regions [29].

We used Principal Component Analysis (PCA) to help interpret the high dimensional data that results from combining multiple texture algorithms and complementary MRI contrasts. Specifically, PCA identifies linear combinations of features, called Principal Components (PCs) that account for most variability across the original features [19–21]. This eliminates >90% of the original features and distills the imaging data to only a few representative PCs for each MRI contrast. This aligns the dimensionality of imaging data with our study’s sample size. Also, we employed three different classification algorithms (DLDA, DQDA, SVM) to help represent a broader spectrum of methodologies that might impact the development and training of our classification model. Of the three algorithms, we found that DLDA provided the greatest test accuracy with the fewest required PCs to build the predictive model.

We recognize potential limitations. First, while this study demonstrates proof of concept that multi-parametric MRI and texture analysis can facilitate tumor localization, we recognize the need to validate these findings in a larger patient population. Second, we classify the adequacy of tumor content based on well-established guidelines, which ensure the quality of DNA isolation and the integrity of genetic analysis. Nonetheless, the requirements for tumor content may change as genetic sequencing technology improves, and future studies may prove necessary to evaluate the accuracy of MRI texture based on other histologic criteria and/or tumor thresholds. Third, image distortions and brain shift following craniotomy could also lead to misregistration errors. To compensate, neurosurgeons used small craniotomy sizes to minimize brain shift and also visually validated stereotactic image location with intracranial neuroanatomic landmarks to help correct for random brain shifts. Rigid-body coregistration of stereotactic and DSC-MR imaging also helped reduce possible geometric distortions [7,16,17]. Overall, our experience suggests combined misregistration at approximately 1–2 mm from both brain shift and registration technique, which is similar to that from previous studies by using stereotactic needle biopsy. Fourth, for each patient, we collected multiple tissue samples from spatially distinct subregions within the same tumor. The vast majority of biopsy targets were separated by >1 cm. While ~10% of samples were separated by 5–10 mm, small ROI sizes minimized the effects of potential sample overlap. Nonetheless, we performed a subanalysis that excluded these minority samples, which demonstrated comparable accuracies to the main study, suggesting negligible impact. A final potential limitation of our study is that the use of multiple biopsies per patient may result in biased estimates, such as an underestimation of the variance of parameter estimates. In previous studies, the impact of using multiple biopsies from the same subject did not impact assessment of the relationship between rCBV and other imaging parameters [16,30]. To address this in the current study, supplemental analysis (**S2 Appendix**) excluded biopsies located within 5-10mm of adjacent biopsies and results remained consistent suggesting a negligible impact of this limitation.

## Supporting Information

S1 Appendix Summary of classification accuracies. Shown are classification accuracies and selected MRI features based on DLDA, DQDA, and SVM classification methods. Incremental accuracy gains are listed for each MRI feature and for each classification method.
(DOCX)

S2 Appendix Summary of tissue samples and test performance from subgroup analysis in training and validation datasets. After excluding 6 biopsy samples (within 5–10 mm of each other) from the training dataset, the table shows the distribution of biopsy samples by tumor content (high- vs. low-) for enhancing core (ENH) and non-enhancing BAT in both training (n = 54) and validation (n = 22) datasets. Test accuracies (sensitivity, specificity) for the optimized model (using the 3 MRI-based features in Table 2) are shown and include positive and negative predictive values (PPV, NPV).
(DOCX)
