# Supplementary material for: Multi-Parametric MRI and Texture Analysis to Visualize Spatial Histologic Heterogeneity and Tumor Extent in Glioblastoma
Source: PLoS One. 2015 Nov 24;10(11):e0141506. doi: 10.1371/journal.pone.0141506 (PMC4658019; doi:10.1371/journal.pone.0141506)
Supplement: S1 Appendix — Shown are classification accuracies and selected MRI features based on DLDA, DQDA, and SVM classification methods. Incremental accuracy gains are listed for each MRI feature and for each classification method. (DOCX) [file pone.0141506.s001.docx]

**S1 Appendix**. **Summary of classification accuracies**.

|  | Selected Features | Accuracy |
| --- | --- | --- |
| DLDA | rCBV-Raw Mean  GLCM-EPI+C  LBP-T1+C | 85% |
| DQDA | DOST-T1+C  rCBV-Raw Mean | 80% |
| SVM | rCBV-Raw Mean | 80% |

Shown are classification accuracies and selected MRI features based on DLDA, DQDA, and SVM classification methods. Incremental accuracy gains are listed for each MRI feature and for each classification method.
