# Supplementary material for: Multi-Parametric MRI and Texture Analysis to Visualize Spatial Histologic Heterogeneity and Tumor Extent in Glioblastoma
Source: PLoS One. 2015 Nov 24;10(11):e0141506. doi: 10.1371/journal.pone.0141506 (PMC4658019; doi:10.1371/journal.pone.0141506)
Supplement: S2 Appendix — After excluding 6 biopsy samples (within 5–10 mm of each other) from the training dataset, the table shows the distribution of biopsy samples by tumor content (high- vs. low-) for enhancing core (ENH) and non-enhancing BAT in both training (n = 54) and validation (n = 22) datasets. Test accuracies (sensitivity, specificity) for the optimized model (using the 3 MRI-based features in Table 2) are shown and include positive and negative predictive values (PPV, NPV). (DOCX) [file pone.0141506.s002.docx]

**S2 Appendix. Summary of tissue samples and test performance from subgroup analysis in training and validation datasets**.

|  | **Training Set** (n = 11 subjects) | | |  | **Validation Set** (n = 7 subjects) | | |  |
| --- | --- | --- | --- | --- | --- | --- | --- | --- |
|  | **ENH** | **BAT** | **Both** |  | **ENH** | **BAT** | **Both** |  |
| **Total samples** | 33 | 21 | 54 |  | 14 | 8 | 22 |  |
| **High tumor samples (% of total samples)** | 21 (63.6%) | 2 (9.5%) | 23 (42.6%) |  | 7 (50%) | 2 (25%) | 9 (41%) |  |
| **Low tumor samples**  **(% of total samples)** | 12 (36.4%) | 19 (90.5%) | 31 (57.4%) |  | 7 (50%) | 6 (75%) | 13 (59%) |  |
| **Imaging accuracy**  **(high vs low tumor)** | 81.8% | 90.5% | 85.2% |  | 78.6% | 87.5% | 81.8% |  |
| **Sensitivity**  **(identify high-tumor)** | 85.7% | 100% | 87% |  | 100% | 100% | 100% |  |
| **Specificity**  **(identify high tumor)** | 75% | 89.5% | 83.9% |  | 57.1% | 83.3% | 69.2% |  |
| **PPV**  **(predict high tumor)** | 85.7% | 50% | 80% |  | 70% | 66.7% | 69.2% |  |
| **NPV**  **(exclude high tumor)** | 75% | 100% | 89.7% |  | 100% | 100% | 100% |  |

After excluding 6 biopsy samples (within 5-10 mm of each other) from the training dataset, the table shows the distribution of biopsy samples by tumor content (high- vs. low-) for enhancing core (ENH) and non-enhancing BAT in both training (n=54) and validation (n=22) datasets. Test accuracies (sensitivity, specificity) for the optimized model (using the 3 MRI-based features in Table 2) are shown and include positive and negative predictive values (PPV, NPV).
